# Path identity as a source of high-dimensional entanglement

**DOI:** 10.1073/pnas.2011405117

**Published:** 2020-10-01

**Authors:** Jaroslav Kysela, Manuel Erhard, Armin Hochrainer, Mario Krenn, Anton Zeilinger

**Affiliations:** ^a^Faculty of Physics, Vienna Center for Quantum Science & Technology, University of Vienna, 1090 Vienna, Austria;; ^b^Institute for Quantum Optics and Quantum Information, Austrian Academy of Sciences, 1090 Vienna, Austria;; ^c^Department of Chemistry & Computer Science, University of Toronto, Toronto, ON M5S 3H6, Canada

**Keywords:** entanglement by path identity, high-dimensional entanglement, path indistinguishability, orbital angular momentum

## Abstract

We present an experimental demonstration of a general entanglement-generation framework, where the form of the entangled state is independent of the physical process used to produce the particles. It is the indistinguishability of multiple generation processes and the geometry of the setup that give rise to the entanglement. Such a framework, termed entanglement by path identity, exhibits a high degree of customizability. We employ one class of such geometries to build a modular source of photon pairs that are high-dimensionally entangled in their orbital angular momentum. We demonstrate the creation of three-dimensionally entangled states and show how to incrementally increase the dimensionality of entanglement. The generated states retain their quality even in higher dimensions. In addition, the design of our source allows for its generalization to various degrees of freedom and even for the implementation in integrated compact devices. The concept of entanglement by path identity itself is a general scheme and allows for construction of sources producing also customized states of multiple photons. We therefore expect that future quantum technologies and fundamental tests of nature in higher dimensions will benefit from this approach.

## Significance

Quantum entanglement amounts to an extremely strong link between two distant particles, a link so strong that it eludes any classical description and so unsettling that Albert Einstein described it as “spooky action at a distance.” Today, entanglement is not only a subject of fundamental research, but also a workhorse of emerging quantum technologies. In our current work we experimentally demonstrate a completely different method of entanglement generation. Unlike many traditional methods, where entanglement arises due to conservation of a physical quantity, such as momentum, in our method it is rather a consequence of indistinguishability of several particle-generating processes. This approach, where each process effectively adds one dimension to the entangled state, allows for a high degree of customizability.

The transition from two- to multidimensional entangled quantum systems brings about radical improvements in the distribution and processing of quantum information. Such systems play an important role in secure high-dimensional superdense coding schemes (1–3); they offer improved noise resistance and increased security against eavesdropping (4, 5); and they are beneficial or even indispensable for fundamental experiments, such as tests of local realism (6–9) or the prospect of teleportation of the entire information stored in a photonic system (10–12). Various degrees of freedom, such as frequency (13), time bin (14–16), and path (17, 18), have been employed so far for the generation of high-dimensionally entangled states. In this paper, we present an experimental proof-of-principle demonstration of a conceptually different framework of generating high-dimensionally entangled states. Multiple spontaneous parametric down-conversion (SPDC) processes are employed, but none of them individually produces entanglement. The entanglement is built in a manner, where not intrinsic properties of a photon-production process, but rather the geometry of the setup governs the structure of the final entangled state. This method amounts to the concept known as entanglement by path identity (19, 20), which was discovered recently with the help of a computer program (21). Utilizing this concept leads to a simple yet versatile design of a source of high-dimensional entanglement. In the following, we present the experimental implementation of this source adapted to the orbital angular momentum (OAM) of photons. Nevertheless, the scheme is not linked to a specific degree of freedom and is valid for other degrees of freedom as well.

The OAM of photons is an in principle unbounded discrete quantity and as such has been used extensively (22–26) to prepare high-dimensionally entangled photonic states. In the traditional way, the OAM-entangled photon pairs are produced in a single SPDC process (27). Albeit convenient, this process exhibits several drawbacks. For example, photon pairs generated in this way have a nonuniform distribution of OAM (28–31). The maximally entangled states can then be generated either by postprocessing techniques, such as Procrustean filtering (32, 33), or by preprocessing of the pump beam. In a recently demonstrated approach (34, 35), a superposition of OAM modes is imprinted by holograms into the pump beam, which translates via down-conversion into maximally entangled states of two photons.

Our technique offers several important advantages over the traditional approach. The source of entangled photon pairs enables us to engineer the state for our needs as both phases and magnitudes in a high-dimensional quantum state can be adjusted completely arbitrarily. One is not limited by the conditions of the employed SPDC processes. This way, various families of states can be produced, such as high-dimensional maximally entangled Bell states that are demanded by applications such as high-dimensional quantum dense coding (36), entanglement swapping (37), or quantum teleportation (38). By proper adjustment of the state’s magnitudes the nonmaximally entangled states maximizing the violation of high-dimensional Bell inequalities (6, 39) can be also produced. The experimental implementation of our source has a modular structure, where adding a single module leads to increasing the entanglement dimension by one. High brightness of our source is possible as all photons are produced already in the desired modes and no photons have to be discarded by postselection.

This work is organized as follows. After a brief introduction to the concept of entanglement by path identity, we describe the experimental design of our source. Then we demonstrate the scalability and versatility of our method by generating several different states in two and in three dimensions. We verify the quality of the produced entangled quantum states using quantum state tomography.

## Entanglement by Path Identity

**Fig. 1.**
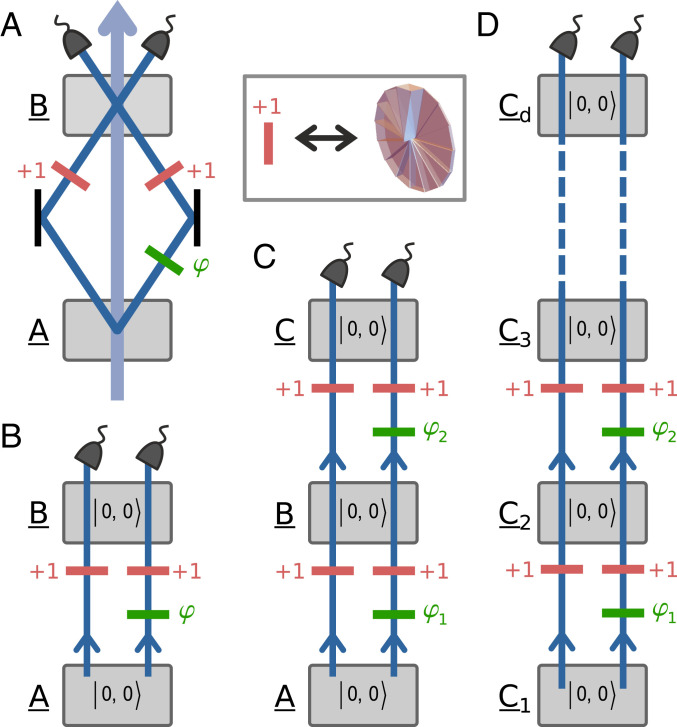
Basic concept. Gray boxes labeled with underlined uppercase letters represent nonlinear crystals, each pumped coherently and each generating with a small probability a pair of photons via an SPDC process. Each generated photon pair is in OAM state $\left|0,0\right\rangle$ with a small contribution of higher-order terms. The two down-converted photons then propagate along their paths in the direction indicated by the arrows and acquire phase shifts $\varphi_{i}$ as well as additional quanta of OAM due to phase and mode shifters. (*A*) The pump beam, represented by an arrow, gives rise to an SPDC process in crystals A and B. Photons generated in crystal A are reflected into crystal B such that their paths are overlapped with paths of photons generated in crystal B. As a consequence, the two coherent SPDC processes in crystals A and B are indistinguishable and the generated photon pairs leave the setup in a two-dimensionally entangled Bell state $1/\sqrt{2}\;\left(\left|0,0\right\rangle +\exp\left(i\varphi \right)\left|1,1\right\rangle \right)$. The quantum of OAM is imparted to the photon by a spiral phase plate shown in *Inset*. (*B*) A schematic picture of the setup in *A*, where the pump beam is not shown. (*C*) The addition of the third crystal to the setup increases the entanglement dimension by one. The resulting state is thus $1/\sqrt{3}\;\left(\left|0,0\right\rangle +\exp\left(i{\bar{\varphi }}_{1}\right)\left|1,1\right\rangle +\exp\left(i{\bar{\varphi }}_{2}\right)\left|2,2\right\rangle \right)$, where ${\bar{\varphi }}_{1}=\varphi_{2}$ and ${\bar{\varphi }}_{2}=\varphi_{1}+\varphi_{2}$. (*D*) One can stack multiple setups from *A* to acquire a series of d crystals that produces a d-dimensionally entangled state $1/\sqrt{d}\left(\left|0,0\right\rangle +\exp\left(i{\bar{\varphi }}_{1}\right)\left|1,1\right\rangle +\cdots +\exp\left(i{\bar{\varphi }}_{d-1}\right)\left|d-1,d-1\right\rangle \right)$, where the relative phases ${\bar{\varphi }}_{i}=\sum_{j=d-i}^{d-1}\varphi_{j}$ are adjusted by an appropriate choice of phase shifters $\varphi_{j}$. The magnitudes of the individual modes are modified by varying the power with which the respective crystals are pumped.

Consider a simple experimental setup consisting of two nonlinear crystals that are aligned in series and coherently emit photons via SPDC, as shown in Fig. 1 *A* and *B*. The pump power for both crystals is set sufficiently low such that events when either crystal emits multiple photon pairs as well as events when both crystals each simultaneously generate a photon pair can be neglected. The propagation paths of the down-converted photons coming from the two crystals are carefully overlapped. As a result, once the photon pair leaves the setup, no information can be obtained, not even in principle, in which crystal the pair was created (40–42). The down-conversion processes in both crystals are adjusted such that photon pairs may be emitted only into the fundamental mode $\left|0,0\right\rangle$ with zero quanta of OAM* . Importantly, no entanglement is generated by either of the two crystals. (In practice, a small contribution of higher-order OAM modes is also present. For the detailed discussion see *SI Appendix*, *Spiral spectrum*.)

Suppose now that two mode shifters are inserted into the setup. These add an extra quantum of OAM to each photon originating in the first crystal and thus act as the only possible source of which-crystal information. As the down-conversion processes in the two crystals are (apart from the OAM) indistinguishable, the resulting state of a detected photon pair is a coherent superposition

$$\left|\psi \right\rangle =\frac{1}{\sqrt{2}}\left(\left|0,0\right\rangle +e^{i\varphi }\left|1,1\right\rangle \right).$$ [1]

In Eq. **1**, φ is the phase between the two SPDC processes imparted by a phase shifter and numbers in ket vectors refer to the OAM quanta of respective photons.

The generation of entangled states as described above is a specific example of the concept termed entanglement by path identity. This concept can be readily generalized for production of high-dimensionally entangled states (19). When the number of crystals in the series is increased to d, and the number of phase and mode shifters is accordingly increased to d−1, high-dimensionally entangled states of the following form are produced as

$$\left|\psi \right\rangle =\overset{d-1}{\underset{\ell =0}{\sum }}c_{\ell }\left|\ell ,\ell \right\rangle ,$$ [2]

where d is the state dimension and $c_{\ell }$ are complex amplitudes (Fig. 1 *C* and *D*). The magnitudes of $c_{\ell }$ can be set by pumping each crystal independently with properly adjusted power. By using different mode shifters for either of the two photons in a down-converted pair, completely arbitrary states can be created. Interestingly, the widely used cross-crystal scheme is the simplest example of the above approach, where two-particle states are entangled in polarization (43–45).

## Setup

The experimental implementation presented here is based on the scheme in Fig. 1*C* with two main modifications. The pump and down-converted beams for each crystal are separated by two Mach–Zehnder interferometers, such that both wavelengths can be manipulated separately. This way, phases as well as magnitudes of individual modes in the quantum state can be adjusted independently. For technical reasons, the down-converted photon pairs were not emitted in a perfectly collinear manner, but had a slight angular deviation of roughly $1^{○}$. This leads to a nonperfect operation of the mode shifter, which functions properly only when both photons propagate through its center. As a countermeasure, we place the mode shifter into the pump beam instead of the down-conversion beam. For details refer to *SI Appendix*, *Detailed setup* and *Coherence conditions*.

**Fig. 2.**
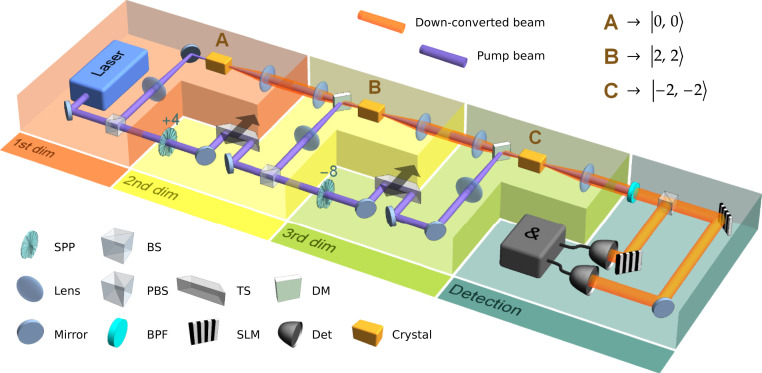
Experimental setup. Three-dimensional states are created by elements in boxes labeled 1st dim, 2nd dim, and 3rd dim. Three periodically poled KTP crystals A, B, and C are pumped with a continuous-wave laser beam at the central wavelength of 405 nm. Frequency-degenerate down-converted photons created by type II collinear SPDC propagate along identical paths into the detection system shown in box Detection. Photons originating in crystal B are created in $\left|2,2\right\rangle$ OAM mode because of a spiral phase plate (SPP +4) inserted after the first beam splitter (BS). In addition, photons originating in crystal C are created in $\left|-2,-2\right\rangle$ mode due to an extra mirror that effectively works as a −8 mode shifter as is explained in the main text. The pump beam is separated from the down-conversion beam by dichroic mirrors (DM) and a band-pass filter (BPF). Before detection, the two down-converted photons are separated on a polarizing beam-splitter (PBS). The state tomography in the OAM degree of freedom is done by projective measurements (27) where specific holograms are projected on two spatial light modulators (SLMs). The reflected photons are subsequently coupled into single-mode fibers and detected by single-photon detectors (Det). The resulting signals are postprocessed by a coincidence counting module (&). The relative phases $\varphi_{1}$ and $\varphi_{2}$ can be adjusted by phase shifters implemented with trombone systems (TS). The magnitudes of individual terms in the quantum state are controlled by setting the splitting ratio of the beam splitters. For the detailed diagram of the experimental setup see *SI Appendix*.

The setup, presented in Fig. 2, was designed to produce three-dimensionally entangled states. Each dimension in the generated quantum state corresponds to one of three nonlinear crystals A, B, or C in the setup. In Fig. 2 this correspondence is emphasized by enclosing the crystals with associated elements into boxes labeled 1st dim, 2nd dim, and 3rd dim. The laser beam is split into three paths to pump each crystal separately. The pump beam for crystal A possesses zero quanta of OAM and so do the down-converted photons, which exit the crystal in state $\left|0,0\right\rangle$. (Apart from the predominant $\left|0,0\right\rangle$ component, also effectively negligible contributions of higher-order OAM terms are present in the photons’ state, as detailed in *SI Appendix*, *Spiral spectrum*.) The pump beam for crystal B acquires four quanta of OAM due to a spiral phase plate (SPP), which is inserted into the beam and plays the role of the mode shifter. Consequently, each down-converted photon generated in crystal B carries two quanta of OAM and the pair is produced in state $\left|2,2\right\rangle$. Similarly, the pump beam for crystal C also acquires four quanta of OAM, but an additional mirror is used to invert the sign of the OAM value from 4 to −4, effectively subtracting eight quanta of OAM. Down-converted photons coming from crystal C are then produced in state $\left|-2,-2\right\rangle$. The resulting quantum state reads

$$\left|\psi \right\rangle =\;\alpha \underset{\text{crystal}\;\text{A}}{\underset{︸}{\left|0,0\right\rangle }}+\beta e^{i\varphi_{1}}\underset{\text{crystal}\;\text{B}}{\underset{︸}{\left|2,2\right\rangle }}+\gamma e^{i\varphi_{2}}\underset{\text{crystal}\;\text{C}}{\underset{︸}{\left|-2,-2\right\rangle }}.$$ [3]

Magnitudes α, β, and γ of the entangled state can be changed by adjusting the relative pump power for each crystal. The relative phases $\varphi_{1}$ and $\varphi_{2}$ are set by positioning two trombone systems that act as phase shifters. By employing only the first two stages of the setup, namely parts in boxes labeled 1st dim and 2nd dim, two-dimensionally entangled states are created. In ref. 46 a similar experimental setup was used to generate three-dimensional (3D) nonentangled states of photons in Fock representation.

We use type II SPDC in all three crystals. To measure the entangled state, we first deterministically separate the two down-converted photons by a polarizing beam splitter. Two spatial light modulators in combination with single mode fibers are used to perform any projective measurement for OAM modes (27). The single photons are then detected by avalanche photon detectors and simultaneous two-photon events are identified by a coincidence logic.

Finally, the resulting quantum states are characterized by complete quantum state tomography. We use a maximum-likelihood reconstruction technique (47) to estimate the physical density matrices of the detected photon pairs. Also, using the fidelity bound derived in ref. 48, the minimum generated entanglement dimensionality is found.

## Experimental Results

The high flexibility of our setup in producing various states is demonstrated in Table 1, where fidelities for different three-dimensionally (and also two-dimensionally) entangled states are presented. These data demonstrate our ability to control the relative phases and magnitudes of the generated quantum states. Most notably, we are able to create three mutually orthogonal and maximally entangled states in three dimensions $\left|\psi_{1}\right\rangle$, $\left|\psi_{2}\right\rangle$, and $\left|\psi_{3}\right\rangle$ with an average fidelity of 87.5±2.2%. These states represent three of nine two-party 3D Bell states, which are important for example in high-dimensional quantum teleportation (38) or high-dimensional superdense coding schemes (1). The orthogonality of these states does not follow directly from the orthogonality of OAM modes, but indeed from differently adjusted phases in the quantum states. Fidelity bounds derived in refs. 48 and 49, which are calculated as a sum of squares of all but the smallest Schmidt coefficients of a given reference state $\left|\psi_{i}\right\rangle$, are used to determine the entanglement dimensionality of the corresponding measured states. When the fidelity F of the experimentally measured density matrix exceeds the associated fidelity bound, the created state is at least three-dimensionally entangled. The presented states $\left|\psi_{1}\right\rangle$ through $\left|\psi_{4}\right\rangle$ are indeed entangled in three dimensions, as their fidelities F satisfy F>2/3≈0.67 and the same is true for $\left|\psi_{5}\right\rangle$ for which F>9/11≈0.82. Likewise, fidelities for two-dimensional (2D) states $\left|\Phi^{+}\right\rangle$ and $\left|\Phi^{-}\right\rangle$ satisfy F>1/2. With the state $\left|\psi_{5}\right\rangle$ we demonstrate the ability to adjust relative magnitudes of terms in the quantum superposition. A nonmaximally entangled state with uneven magnitudes, very similar to $\left|\psi_{5}\right\rangle$, provides the maximal violation of the 3D generalization of the Bell inequalities (6, 39). The generation rates of our setup are around 1,200 Hz for the 3D states and around 1,400 Hz for the 2D states. It is important to mention here that these count rates are the actually detected ones. All losses from the detection scheme, such as spatial light modulators, detectors, and other optical elements, are already included. With the constant total pump power the two rates should be equal. The reason why the former is smaller is that an SPDC process is less efficient when pumped by a beam with a nonzero number of OAM quanta, as is the case for crystals B and C. This effect is not present when all crystals are pumped by a fundamental mode as proposed in the scheme in Fig. 1.

**Table 1.** Fidelities $F\left(\left|\psi \right\rangle ,\rho \right)=\text{Tr}\left(\left|\psi \right\rangle \left\langle \psi \right|\rho \right)$ between several two- and three-dimensionally entangled states $\left|\psi \right\rangle$ and their experimental realizations ρ

State	Fidelity F
$\left|\Phi^{+}\right\rangle =1/\sqrt{2}\left(\left|0,0\right\rangle +\left|2,2\right\rangle \right)$	0.904±0.005
$\left|\Phi^{-}\right\rangle =1/\sqrt{2}\left(\left|0,0\right\rangle -\left|2,2\right\rangle \right)$	0.891±0.005
$\left|\psi_{1}\right\rangle =\frac{1}{\sqrt{3}}\left(\left|0,0\right\rangle +\left|2,2\right\rangle +\left|-2,-2\right\rangle \right)$	0.870±0.005
$\left|\psi_{2}\right\rangle =\frac{1}{\sqrt{3}}\left(\left|0,0\right\rangle +\omega \left|2,2\right\rangle +\omega^{-1}\left|-2,-2\right\rangle \right)$	0.852±0.007
$\left|\psi_{3}\right\rangle =\frac{1}{\sqrt{3}}\left(\left|0,0\right\rangle +\omega^{-1}\left|2,2\right\rangle +\omega \left|-2,-2\right\rangle \right)$	0.903±0.006
$\left|\psi_{4}\right\rangle =\frac{1}{\sqrt{3}}\left(\left|0,0\right\rangle -\left|2,2\right\rangle -\left|-2,-2\right\rangle \right)$	0.890±0.004
$\left|\psi_{5}\right\rangle =\frac{1}{\sqrt{22}}\left(2\left|0,0\right\rangle +3\left|2,2\right\rangle +3\left|-2,-2\right\rangle \right)$	0.848±0.008

States $\left|\psi_{1}\right\rangle$, $\left|\psi_{2}\right\rangle$, and $\left|\psi_{3}\right\rangle$ form an orthonormal set of maximally entangled states in three dimensions $\omega =e^{2\pi i/3}$. State $\left|\psi_{5}\right\rangle$ is a manifestation of our ability to control not only relative phases in the quantum state, but also relative magnitudes. The error estimates are calculated by propagation of Poissonian statistics of coincidence counts and do not take into account possible systematic errors. For detailed discussion of experimental data refer to *SI Appendix*, *State tomography results*.

**Fig. 3.**
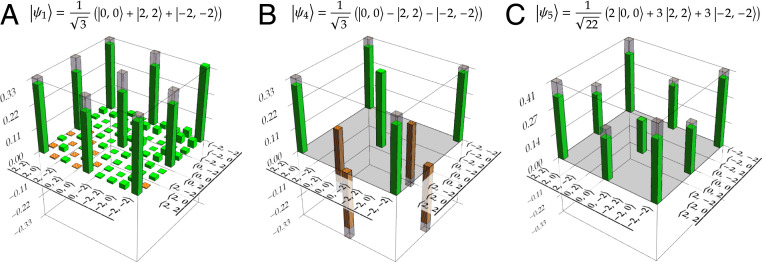
Examples of the three-dimensionally entangled states $\left|\psi_{1}\right\rangle$, $\left|\psi_{4}\right\rangle$, and $\left|\psi_{5}\right\rangle$ produced (Table 1). With our method we can control the relative phases, as demonstrated in *A* and *B*, as well as relative magnitudes, as shown in *C*. Only real parts are shown; imaginary parts lie in the range (−0.12,0.12) for all cases. Background noise contributions lie in the range (−0.04,0.04) for all cases and are explicitly shown only in *A*. This background is omitted in *B* and *C* to improve readability. Green (solid) and orange (hatched) bars represent positive and negative values of reconstructed density matrices, respectively. Gray translucent bars represent the theoretical expectation. Fidelities of the measured states with their reference states are 87.0±0.5%, 89.0±0.4%, and 84.8±0.8%, respectively.

The real parts of the density matrices for three of the states presented in Table 1 are displayed in Fig. 3. There, the measurement results (solid bars) are compared to the theoretical expectations (translucent bars). The average fidelity 87.3±2.2% of three-dimensionally entangled states does not decrease significantly when compared to the average fidelity of 89.8±0.9% of 2D states. The quality of the entangled states is thus mostly unaffected when going from two to three dimensions and indicates that our approach can be feasible for even higher dimensions. The fidelities reported in Table 1 do not reach unity for two main reasons. First, imperfect coherence of the SPDC processes amounts to roughly 5% decrease in the fidelities for all reported states irrespective of their dimensionality. The main limitation for achieving higher coherence is slight distinguishability of the SPDC sources, which we attribute to small differences in the spectral and polarization degrees of freedom of the down-converted photons. Second, an imprecise setting of local phases, slight misalignment, and the presence of higher-order OAM modes lead to an extra decrease of fidelities, which varies for different states. This explains the range 85 to 90% of fidelities for different 3D states. Nevertheless, none of these imperfections are of fundamental nature. We analyze the causes of these imperfections in detail in *SI Appendix* and therefore facilitate technical improvements in future development iterations. Complete state tomography data are presented in *SI Appendix*, *State tomography results*.

## Alternative Designs

The modular structure of the setup gives rise to the scalability of our scheme in the sense that to increase the entanglement dimension by one requires a mere addition of a single crystal and a single mode shifter (SPP). To further improve the performance, some modifications to our experimental implementation can be made. We adopted the Mach–Zehnder interferometric configuration in our experiment. This gives us freedom to access and manipulate the pump and down-conversion beams separately with no need of custom-made components. The distance between two successive crystals in our current setup is 600 mm. Due to these large interferometers, active stabilization is inevitable. However, scaling down the distances and employing integrated fabrication techniques as used in microchip fabrication lead to significantly more stable interferometers. An alternative approach is to circumvent interferometers completely by, for example, using wavelength-dependent phase shifters and q plates (50, 51), which is inherently stable.

The framework of entanglement by path identity can be easily employed to generate hyperentangled states. Our source of photon pairs can be modified to produce polarization–OAM hyperentanglement when the noncollinear type II SPDC process is utilized in each crystal. This way, the two photons are already created in a polarization-entangled state and due to the geometry of the setup they become also entangled in OAM. In addition, the framework represents a more efficient alternative to traditional techniques to generate multipartite entanglement (19). For instance, in the case of the 3D three-photon Greenberger–Horne–Zeilinger (GHZ) state, the design based on the entanglement by path identity produces entangled states with probability that is eight times larger than when one uses the traditional approach based on interference (12).

## Conclusion

We performed a proof-of-principle experiment of a method called path identity to generate high-dimensionally entangled quantum states. In contrast to previous entanglement creation schemes, here the form of the created quantum state is not dependent on the photon pair creation process itself, but the geometrical arrangement of the setup. Besides its conceptual difference, our approach has two core strengths: a simple and a modular design. The simple geometry-based approach allows us to design an experimental layout that creates versatile and high-dimensionally entangled photon pairs in OAM. These states can be readily utilized in various applications such as superdense coding, high-dimensional quantum teleportation, and violations of generalized Bell inequalities.

We confirmed the modularity of our source by generating different entangled quantum states in two and three dimensions. Thereby we found that the average fidelity of the created states is not decreasing significantly. Thus we believe that extending this modular arrangement is possible and will lead to even higher-dimensionally entangled states in the future. Another very appealing feature of our method is that different families of spatial modes can be used. It is, therefore, possible to create high-dimensional entangled photon pairs in specific modes optimized for free-space communication or even fiber-based systems.

## Supplementary Material

Supplementary File

## Data Availability

All study data are included in this article and *SI Appendix*.

## Footnotes

*Parameters of the two SPDC processes are chosen such that photon properties such as frequency, polarization, and OAM are identical for both crystals and also higher-order OAM modes are highly suppressed.
