# Supplementary material for: Path identity as a source of high-dimensional entanglement
Source: Proc Natl Acad Sci U S A. 2020 Oct 1;117(42):26118–22. doi: 10.1073/pnas.2011405117 (PMC7584901; doi:10.1073/pnas.2011405117)
Supplement: Supplementary File [file pnas.2011405117.sapp.pdf]

PNAS
www.pnas.org

Supplementary Information for

Path Identity as a Source of High-Dimensional Entanglement

Jaroslav Kysela, Manuel Erhard, Armin Hochrainer, Mario Krenn and Anton Zeilinger

Jaroslav Kysela, Manuel Erhard, Anton Zeilinger
E-mail: jaroslav.kysela@univie.ac.at, manuel.erhard@univie.ac.at, anton.zeilinger@univie.ac.at

This PDF file includes:

Figs. S1 to S8
Table S1
SI References

www.pnas.org/cgi/doi/10.1073/pnas.2011405117

Detailed setup

The simplified scheme of the experimental setup shown in Fig. 2 in the main text does not show the implementation of the variable splitting-ratio beam-splitters, phase shifters, and the interferometer stabilization system. In Fig. S1 the detailed scheme of our setup can be found. The variable splitting-ratio beam-splitter is built from a polarizing beam-splitter and two half-wave plates. After each beam-splitter both beams possess horizontal polarization.

To phase-stabilize the interferometer in the setup, a light beam emitted from an additional laser at the central wavelength of 710 nm is injected into the unused input port of the beam-splitter. The two created spatial modes of light are recombined at the dichroic mirror and the resulting interfering beam is monitored by a fast photodiode. The detection signal is processed by a PID controller and a feedback signal is fed to the piezo-actuator-driven mirror in order to compensate for phase fluctuations. For comparison, in Fig. S2 the time dependence of the coincidence count rate with and without stabilization is presented. This active feedback loop stabilization system also works as a fine phase shifter, for details see section "Phase adjustment." The coarse adjustment of the phase can be done by a trombone system built in one arm of the interferometer.

The OAM mode shifter is inserted into the pump beam instead of the down-converted beams, as suggested by the principle scheme in Fig. 1 in the main text, for the following reason. Requirements on the temperature of nonlinear crystals and on the frequencies of down-converted photons did not allow for perfect collinearity of the generated pairs. In order to operate optimally the mode shifter has to be precisely centered with respect to the beam it acts upon. The failure to satisfy this condition for both photons of the pair leads to generation of undesirable higher-order OAM terms and a spread of the resulting OAM spiral spectrum.

Phase adjustment

Relative phases in generated quantum states can be tuned precisely by a series of quarter-, half-, and quarter-wave plates, henceforth referred to as the QHQ scheme, that are inserted into the locking-laser beam as shown in Fig. S1. The QHQ scheme manipulates the local phase between the horizontal and vertical polarization components of the locking-laser beam. At the polarizing beam-splitter the relative phase between polarizations translates into relative phase between the two modes of propagation of the locking-laser beam through the interferometer. After recombination of the two paths at the dichroic mirror the intensity of the interfering beam is measured by a photodiode, which feeds the measured signal to the PID controller. The controller interprets the intensity change as unwanted fluctuation and offsets the piezo actuator to compensate for it. This way the phase change is imprinted into the pump beam and therefore into the down-converted photons as well.

When quarter-wave plates in the QHQ scheme are rotated correctly, the middle half-wave plate alone can be turned to adjust conveniently the phase in generated quantum states. In what follows, the working principle of QHQ scheme is explained.

In Jones matrix formalism, a quarter-wave plate (Q) and a half-wave plate (H), rotated through angle α with respect to the vertical direction, are represented by

$$Q(\alpha) = R(\alpha) \begin{pmatrix} 1 & 0 \\ 0 & i \end{pmatrix} R(-\alpha), \quad [1]$$

$$H(\alpha) = R(\alpha)\, \sigma_Z\, R(-\alpha) \quad [2]$$

respectively, where $R(\alpha)$ is a rotation matrix and σ_Z is Pauli-Z matrix. Their forms read

$$R(\alpha) = \begin{pmatrix} \cos(\alpha) & -\sin(\alpha) \\ \sin(\alpha) & \cos(\alpha) \end{pmatrix}, \; \sigma_Z = \begin{pmatrix} 1 & 0 \\ 0 & -1 \end{pmatrix}. \quad [3]$$

We first explain the working principle of the QHHQ scheme, where two half-wave plates are used, and then show that this scheme is equivalent to the QHQ scheme. It can be shown that a single Q and a single H can be used to transform any elliptical polarization into a linear polarization. Such a linear polarization can then be easily rotated by a half-wave plate independently of the input polarization. Finally, a quarter-wave plate rotated through $\pi/4$ transforms a linearly polarized state with polarization angle φ into an equally-weighted superposition of horizontal and vertical polarization components as

$$Q\left(\frac{\pi}{4}\right) \begin{pmatrix} \cos(\varphi) \\ \sin(\varphi) \end{pmatrix} = \frac{e^{i(\frac{\pi}{4}-\varphi)}}{\sqrt{2}} \begin{pmatrix} 1 \\ e^{i(2\varphi-\frac{\pi}{2})} \end{pmatrix}. \quad [4]$$

The polarization angle φ is therefore transformed into a relative phase. In total, $Q(\frac{\pi}{4})H(\alpha)H(\beta)Q(\gamma)$ scheme allows one to obtain a beam with polarization of the form $H + e^{i\omega}V$, where β and γ depend on the input polarization as generated by the locking laser and relative phase ω depends effectively only on the rotation angle α of the half-wave plate.

It is straightforward to prove two useful relations $H(\alpha)H(\beta) = H(\alpha-\beta)\sigma_Z$ and $\sigma_Z Q(-\gamma) = Q(\gamma)\sigma_Z$, so that

$$Q\left(\frac{\pi}{4}\right) H(\alpha)H(\beta)Q(\gamma) = Q\left(\frac{\pi}{4}\right) H(\alpha-\beta)Q(-\gamma)\sigma_Z. \quad [5]$$

The extra σ_Z merely shifts the relative phase of the incoming beam by π, which is corrected for by the proper setting of β and γ. We thus showed that $Q(\frac{\pi}{4})H(\alpha-\beta)Q(-\gamma)$ scheme can be used to adjust the relative phase in the state of the locking-laser beam by turning the half-wave plate appropriately.

Coherence conditions

The generation of quantum states via the concept of entanglement by path identity requires coherent and indistinguishable photon-creation processes. To verify a sufficient level of coherence in our setup, the spiral phase plate was removed from the setup in Fig. S1 and the interference between different SPDC processes in the zero OAM mode was measured. The quality of the coherence is quantified by the interferometric visibility $V = (\mathrm{Max}(D) - \mathrm{Min}(D))/(\mathrm{Max}(D) + \mathrm{Min}(D))$, where D is the coincidence count rate. Results for crystals A and B are shown in Fig. S3. The observed visibility exceeds 97 % in this case and the two SPDC processes in crystals A and B thus exhibit a high degree of coherence. Analogous results were also obtained when SPDC processes in crystals B and C were interfering.

In general, the following relation has to be satisfied in order to observe interference for collinear SPDC processes. Let L_{coh} be the coherence length of the pump laser, which is in our case greater than 2 cm. Moreover, let $L_{\mathrm{p,A}}$ and $L_{\mathrm{p,B}}$ be the distances traveled by the pump beam from the beam splitter to crystals A and B, respectively. The physical conditions for coherence of corresponding SPDC processes are then given by (1, 2)

$$|L_{\mathrm{p,B}} - L_{\mathrm{p,A}} - L_{\mathrm{SPDC}}| \leq L_{\mathrm{coh}}, \qquad [6]$$

where L_{SPDC} is the propagation distance of down-converted photons from crystal A to crystal B. In other words, the optical path length difference between the two arms of the interferometer must be within the coherence length of the pump laser.

Spiral spectrum

Typically, the state of photon pairs $|\psi\rangle$ produced in an SPDC process contains a non-negligible admixture of higher-order OAM terms

$$|\psi\rangle = \alpha_0|0,0\rangle + \alpha_1(|1,-1\rangle + |-1,1\rangle) + \alpha_2(|2,-2\rangle + |-2,2\rangle) + \dots \qquad [7]$$

Magnitudes of these contributions in general decrease for increasing OAM order. The precise relationship between the OAM order k and its complex amplitude α_k is governed by several tunable parameters (3). In order for our scheme, presented in the main text, to work properly, these parameters have to be chosen such that all higher-order OAM terms coming from the SPDC processes are significantly suppressed. As shown in Fig. S4 for crystal A, we were able to suppress the probability $|\alpha_1|^2$ of detecting the photons in the first OAM order below five percent of the probability of detecting them in the zero mode $|\alpha_0|^2$. Similar results were obtained for crystals B and C as well. These data justify our assumption in the main text that SPDC process produces photons only in their zero OAM mode.

State tomography results

The real and imaginary parts of density matrices corresponding to states presented in Tab. I and Fig. 3 in the main text are shown Fig. S5 for the two-dimensional states and in Fig. S6 for the three-dimensional states (see also the first and second columns of Tab. S1).

There are two kinds of undesirable effects that result in imperfect fidelities between the desired states and their experimental realizations as reported in Tab. I in the main text. The first type are incoherent admixtures that lead to mixed-state contributions in our data. Their source is slight distinguishability of SPDC processes in the three non-linear crystals. When an optimization algorithm is run to find a pure state that maximizes its overlap with the reconstructed density matrix, it is possible to determine the magnitude of the mixed part in the quantum state. In the third column of Tab. S1 the fidelities $F(|\rho_i\rangle, \rho_i)$ of such optimal pure states $|\rho_i\rangle$ with the reconstructed states ρ_i are presented. For two-dimensional states the average mixed contribution amounts to the decrease of roughly five percent as the average fidelity is in this case 0.946 ± 0.005. For three-dimensional states we get the average fidelity of 0.945 ± 0.005 demonstrating that coherence of SPDC processes is not really affected when the third crystal is added to the setup. The complex coefficients for individual optimal states $|\rho_i\rangle$ are plotted in a complex plane in Fig. S7 for two-dimensional states and in Fig. S8 for three-dimensional states.

In the forth column of Tab. S1 the fidelities between optimal pure states $|\rho_i\rangle$ and the corresponding reference states $|\psi_i\rangle$ are shown. These values indicate the amount of the second, coherent, type of undesirable effects introduced into our experimentally obtained states. The coherent discrepancies result from: (A) non-negligible presence of higher-order OAM modes and (B) from imprecise adjustment of phases and magnitudes of the quantum state. To see the effect of either of the coherent errors individually, in the fifth and sixth columns of Tab. S1 the two scenarios are presented. States $|\rho_i^{(A)}\rangle$ are obtained by taking the renormalized optimal state $|\rho_i\rangle$ from which all undesirable higher-order OAM terms are removed. The fidelity between such a state and the reference state shows how significant the imprecise setting of the phases (and magnitudes) of the desired terms is. On the other hand, states $|\rho_i^{(B)}\rangle$ are obtained from the optimal states $|\rho_i\rangle$ with the only modification that the phases of desired terms are corrected to their expected values. The fidelity between state $|\rho_i^{(B)}\rangle$ and its corresponding reference state then demonstrates significance of the higher-order OAM contributions in the quantum state. By inspection of the table one sees that both (A) and (B) play roughly equally important role when the differences between the optimized pure state and the reference state are considered.

To reduce both the higher-order OAM contributions and the discrepancy in the phase settings more precise adjustment of waveplates and trombone systems in our setup as well as more advanced spiral spectrum customization of the non-linear crystals are necessary.

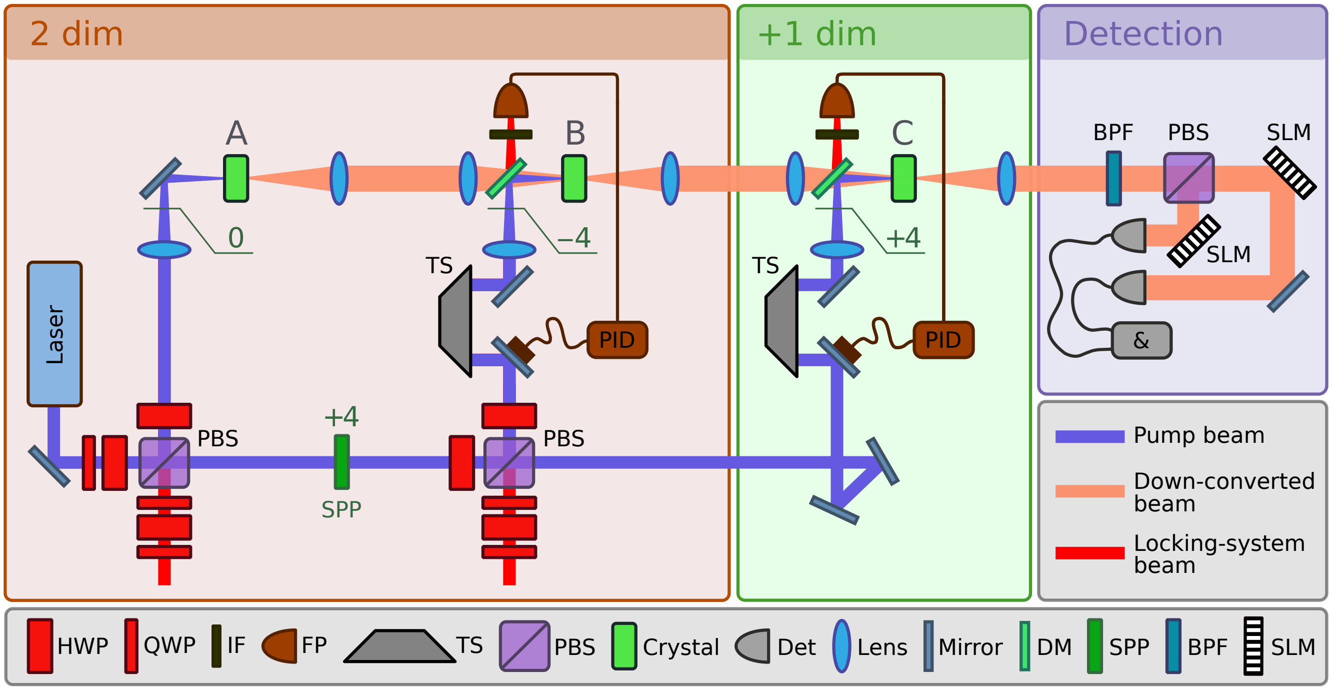

Fig. S1. Detailed setup. For a description of the basic setup see Fig. 2 in the main text. The two interferometers are phase-locked by active feedback systems. An additional laser diode with central wavelength 710 nm is used to provide a locking-system beam that is injected into unused ports of beam-splitters and leaves the setup through dichroic mirrors (DM). After filtering out the pump beam by an interference filter (IF), the interference fluctuations of the locking-laser beam are detected by a fast photodiode (FP). The obtained signal is processed by a PID controller and a feedback signal is sent to a piezo actuator attached to one of the mirrors in the interferometer. The relative phases of the down-converted beams (denoted by φ_1 and φ_2 in the main text) can be adjusted by trombone systems (TS) and by a proper setting of polarization of the corresponding locking-system beam. This is accomplished by a series of two quarter-wave plates (QWP) and one half-wave plate (HWP) as is explained in the text. Magnitudes of individual terms in the quantum state are controlled by setting the splitting ratio of the beam-splitters. A variable splitting-ratio beam-splitter is implemented by a polarizing beam-splitter (PBS) with two half-wave plates. All three crystals are 10 mm long ppKTP non-linear crystals. Det – single photon detector, SPP – spiral phase plate, BPF – band-pass filter, SLM – spatial light modulator.

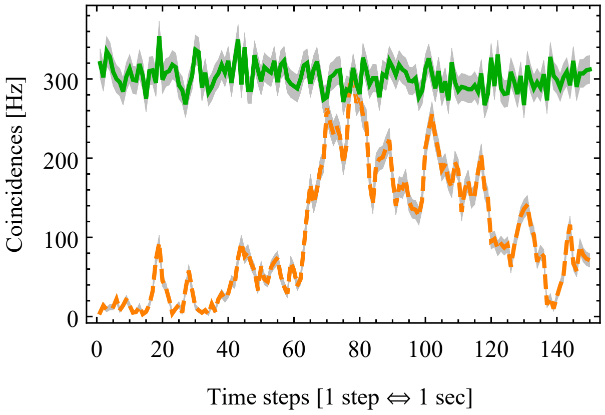

Fig. S2. Comparison of coincidence-count signal fluctuations for the case when the interferometer in the setup is actively stabilized (green solid line) and when it is not (orange dashed line). Photon pairs coming from crystals A and B, pumped with a beam having zero quanta of OAM, were collected in time steps of one second. Gray shaded areas correspond to one standard deviation region of collected data when Poissonian counting statistics is assumed.

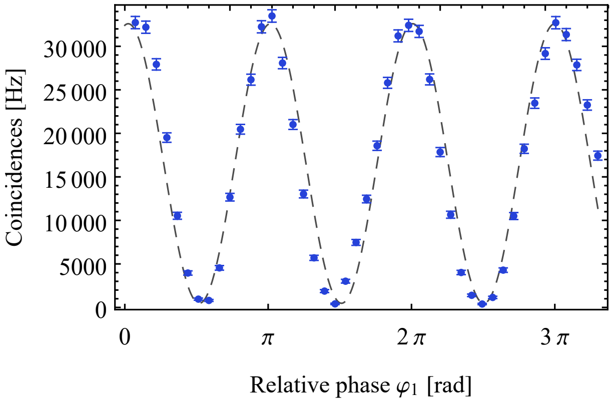

Fig. S3. Interference of two SPDC processes in crystals A and B in the fundamental OAM mode $|0,0\rangle$. Coincidence counts are collected while the relative phase φ_1 is changed. Errors are determined assuming Poissonian counting statistics. The dashed line represents the fit of the displayed data. The obtained visibility is $97.1 \pm 0.5\%$.

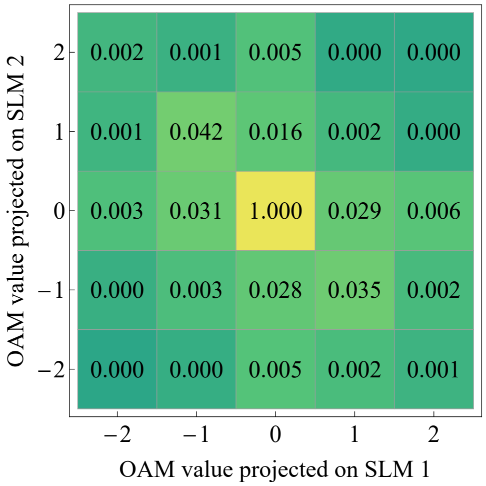

Fig. S4. Normalized coincidences for different projective measurements of OAM in the computational basis. Photon pairs produced in crystal A, pumped with a beam having zero quanta of OAM, were detected and coincidence counts collected for different choices of projections. On the two SLMs, see the detection system in Fig. S1, the wavefronts corresponding to OAM modes $-2, \ldots, 2$ were projected. In the ideal case, only diagonal entries would be nonzero. The coincidence rate for $|0, 0\rangle$ mode is more than twenty times higher than the next highest coincidence rate.

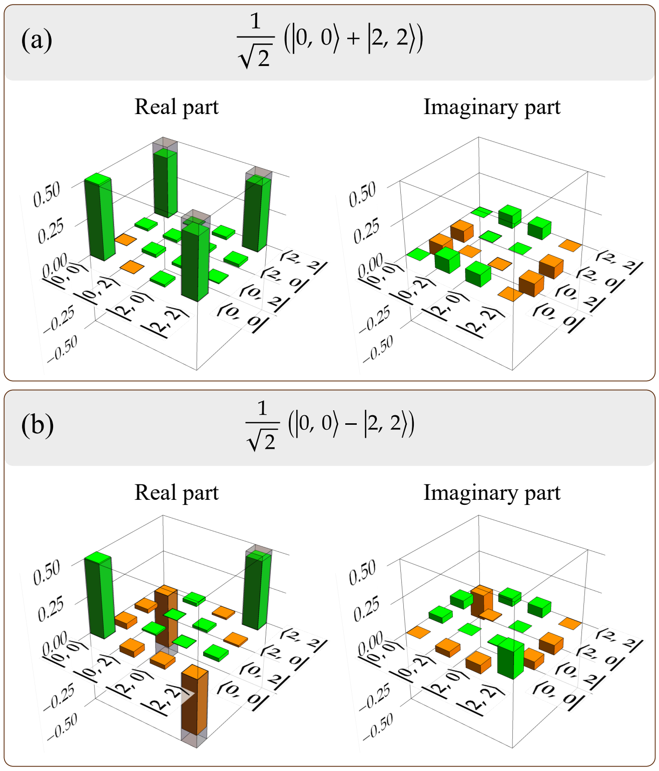

Fig. S5. Two selected two-dimensionally entangled states. Real and imaginary parts are shown. Green and orange bars represent positive and negative values of reconstructed density matrices, respectively. Gray bars represent the theoretical expectation. (a) State $|\Phi^+\rangle = 1/\sqrt{2}(|0,0\rangle + |2,2\rangle)$ with fidelity $90.4 \pm 0.5\%$. (b) State $|\Phi^-\rangle = 1/\sqrt{2}(|0,0\rangle - |2,2\rangle)$ with fidelity $89.1 \pm 0.5\%$.

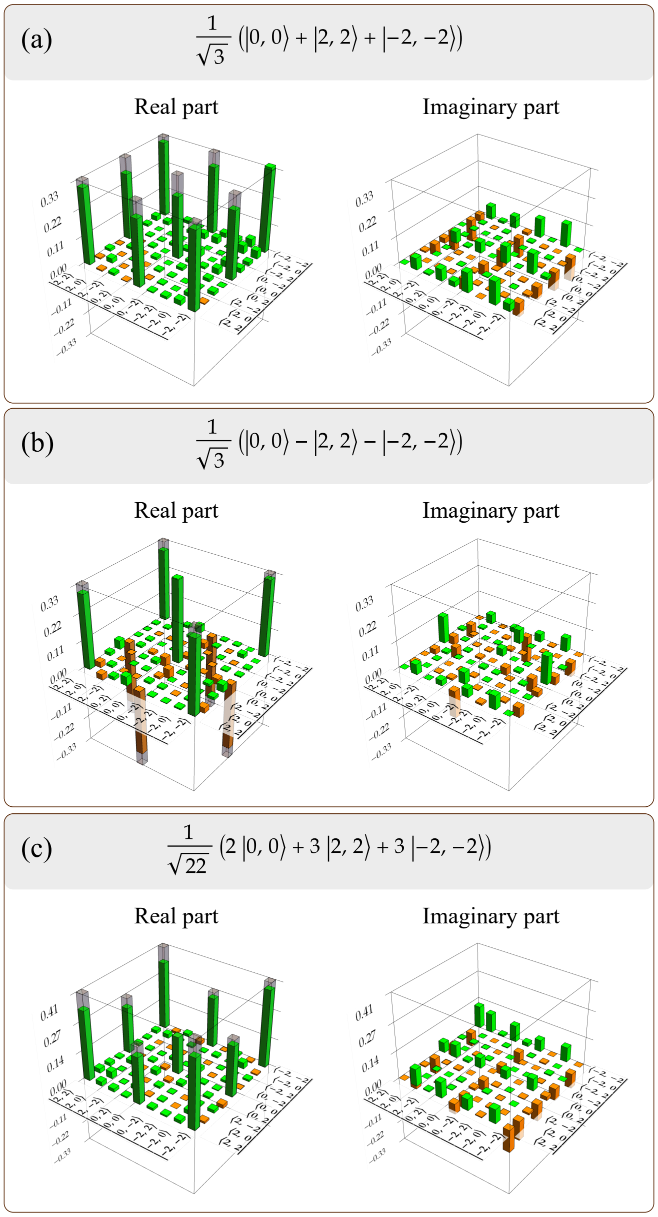

Fig. S6. Three selected three-dimensionally entangled states. Real and imaginary parts of density matrices are shown. Green and orange bars represent positive and negative values of reconstructed density matrices, respectively. Gray bars represent the theoretical expectation. Fidelities of the measured states with their reference states are $87.0 \pm 0.5\%$, $89.0 \pm 0.4\%$ and $84.8 \pm 0.8\%$, respectively. (a) State $|\psi_1\rangle = 1/\sqrt{3}(|0,0\rangle + |2,2\rangle + |-2,-2\rangle)$. (b) State $|\psi_4\rangle = 1/\sqrt{3}(|0,0\rangle - |2,2\rangle - |-2,-2\rangle)$. (c) State $|\psi_5\rangle = 1/\sqrt{22}(2|0,0\rangle + 3|2,2\rangle + 3|-2,-2\rangle)$.

Table S1. Fidelities $F(|\psi_i\rangle, \rho_i) = \mathrm{Tr}(|\psi_i\rangle\langle\psi_i|\rho_i)$ between several two- and three-dimensionally entangled states $|\psi_i\rangle$ and their experimental realizations ρ_i together with the analysis of different sources of imperfections.

Reference state $\lvert\psi_i\rangle$	$F(\lvert\psi_i\rangle, \rho_i)$	$F(\lvert\rho_i\rangle, \rho_i)$	$F(\lvert\psi_i\rangle, \lvert\rho_i\rangle)$	$F(\lvert\psi_i\rangle, \lvert\rho_i^{(A)}\rangle)$	$F(\lvert\psi_i\rangle, \lvert\rho_i^{(B)}\rangle)$
$\lvert\Phi^+\rangle = 1/\sqrt{2}(\lvert 0,0\rangle + \lvert 2,2\rangle)$	0.904 ± 0.005	0.942 ± 0.005	0.960	0.999	0.960
$\lvert\Phi^-\rangle = 1/\sqrt{2}(\lvert 0,0\rangle - \lvert 2,2\rangle)$	0.891 ± 0.005	0.950 ± 0.005	0.936	0.954	0.980
$\lvert\psi_1\rangle = \frac{1}{\sqrt{3}}(\lvert 0,0\rangle + \lvert 2,2\rangle + \lvert -2,-2\rangle)$	0.870 ± 0.005	0.946 ± 0.005	0.919	0.994	0.921
$\lvert\psi_2\rangle = \frac{1}{\sqrt{3}}(\lvert 0,0\rangle + \omega\lvert 2,2\rangle + \omega^{-1}\lvert -2,-2\rangle)$	0.852 ± 0.007	0.954 ± 0.006	0.891	0.920	0.969
$\lvert\psi_3\rangle = \frac{1}{\sqrt{3}}(\lvert 0,0\rangle + \omega^{-1}\lvert 2,2\rangle + \omega\lvert -2,-2\rangle)$	0.903 ± 0.006	0.944 ± 0.005	0.961	0.974	0.986
$\lvert\psi_4\rangle = \frac{1}{\sqrt{3}}(\lvert 0,0\rangle - \lvert 2,2\rangle - \lvert -2,-2\rangle)$	0.890 ± 0.004	0.950 ± 0.004	0.938	0.967	0.970
$\lvert\psi_5\rangle = \frac{1}{\sqrt{22}}(2\lvert 0,0\rangle + 3\lvert 2,2\rangle + 3\lvert -2,-2\rangle)$	0.848 ± 0.008	0.934 ± 0.006	0.907	0.978	0.927

First two columns reproduce the data in Tab. I in the main text. The third column demonstrates the role of incoherent admixture into our experimentally measured states. On average, roughly five percent in fidelities is lost due to distinguishability of the SPDC sources, irrespective of a given quantum state. The fidelities between the pure part of a measured quantum states and the associated reference states is shown in the forth column. These fidelities vary between 0.891 and 0.961 due to two main types of undesirable coherent effects. The role of imprecisely adjusted phases in the quantum state is demonstrated in the fifth column with auxiliary states $|\rho_i^{(A)}\rangle$, whereas the significance of unwanted higher OAM terms is illustrated in the sixth column with states $|\rho_i^{(B)}\rangle$. It follows that both kinds of coherent imperfections are present in the experimental data. The experimental error estimates in the second and third columns are calculated by propagation of Poissonian statistics of coincidence counts and do not take into account possible systematic errors; $\omega = e^{2\pi i/3}$.

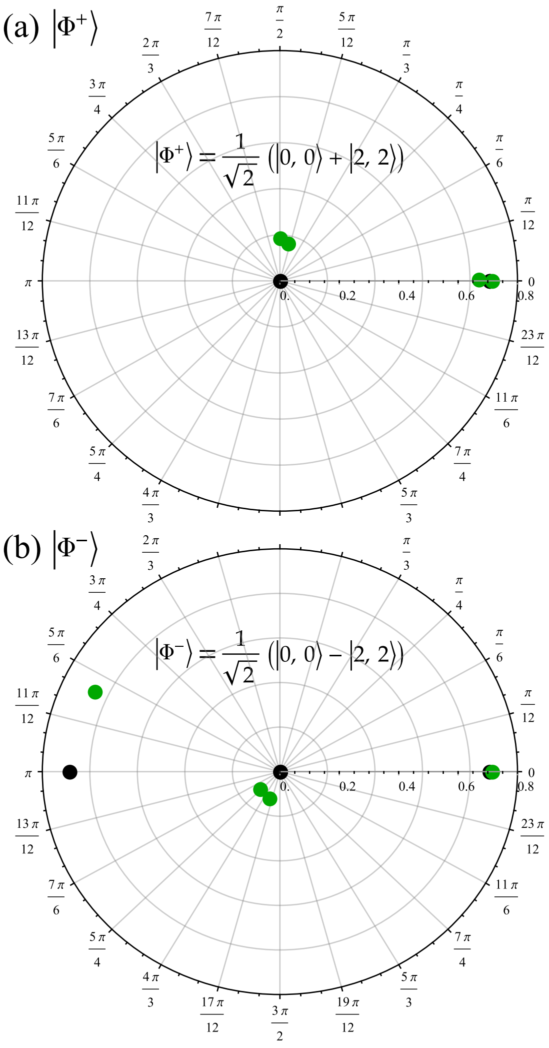

Fig. S7. Complex coefficients of pure states $|\rho_i\rangle$ (green dots) maximizing the overlap with measured density matrices ρ_i and corresponding reference states $|\psi_i\rangle$ (black dots) in two dimensions. The radial coordinate corresponds to the magnitude of the coefficient and the angular coordinate to its phase. (a) State $|\Phi^+\rangle = 1/\sqrt{2}(|0,0\rangle + |2,2\rangle)$. (b) State $|\Phi^-\rangle = 1/\sqrt{2}(|0,0\rangle - |2,2\rangle)$.

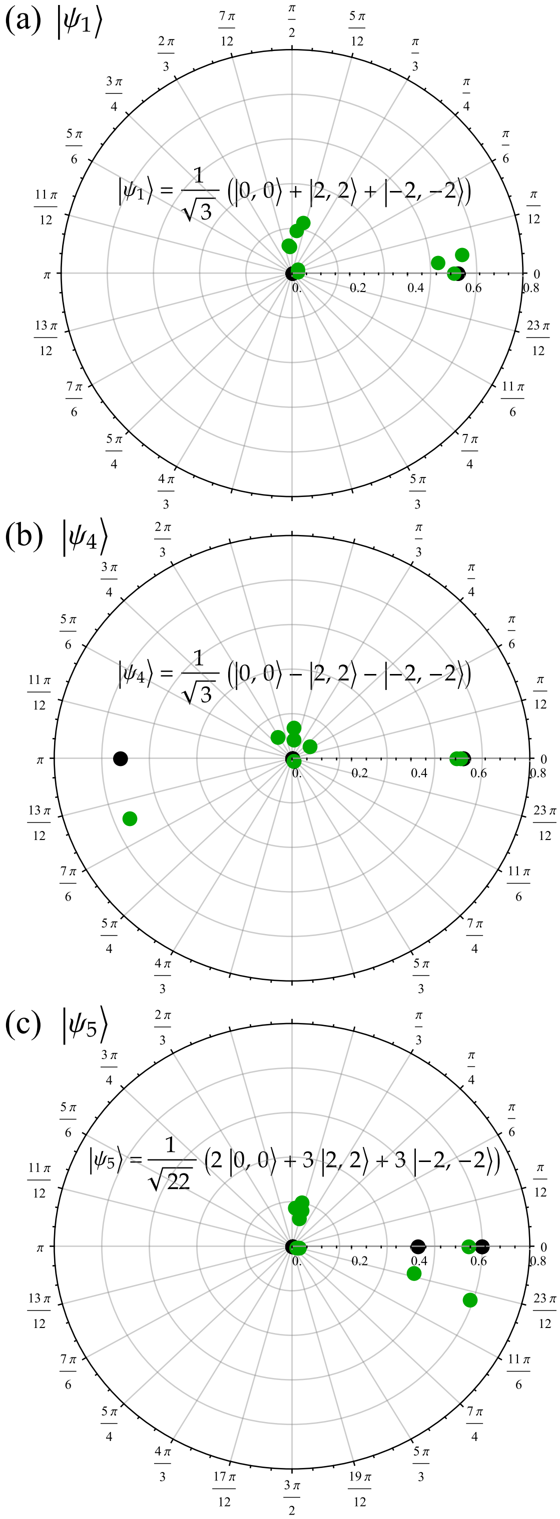

Fig. S8. Complex coefficients of pure states $|\rho_i\rangle$ (green dots) maximizing the overlap with measured density matrices ρ_i and corresponding reference states $|\psi_i\rangle$ (black dots) in three dimensions. The radial coordinate corresponds to the magnitude of the coefficient and the angular coordinate to its phase. (a) State $|\psi_1\rangle = 1/\sqrt{3}(|0,0\rangle + |2,2\rangle + |-2,-2\rangle)$. (b) State $|\psi_4\rangle = 1/\sqrt{3}(|0,0\rangle - |2,2\rangle - |-2,-2\rangle)$. (c) State $|\psi_5\rangle = 1/\sqrt{22}(2|0,0\rangle + 3|2,2\rangle + 3|-2,-2\rangle)$.

References

1. AK Jha, MN O'Sullivan, KWC Chan, RW Boyd, Temporal coherence and indistinguishability in two-photon interference effects. *Phys. Rev. A* **77**, 021801 (2008).
2. PH Souto Ribeiro, GA Barbosa, Mirror effects and induced coherence in parametric down-conversion. *Opt. Commun.* **139**, 139–147 (1997).
3. FM Miatto, et al., Bounds and optimisation of orbital angular momentum bandwidths within parametric down-conversion systems. *The Eur. Phys. J. D* **66**, 178 (2012).
